# Sex bias in celiac disease: XWAS and monocyte eQTLs in women identify *TMEM187* as a functional candidate gene

**DOI:** 10.1186/s13293-023-00572-1

**Published:** 2023-12-11

**Authors:** Alba Hernangomez-Laderas, Ariadna Cilleros-Portet, Silvia Martínez Velasco, Sergi Marí, María Legarda, Bárbara Paola González-García, Carlos Tutau, Iraia García-Santisteban, Iñaki Irastorza, Nora Fernandez-Jimenez, Jose Ramon Bilbao

**Affiliations:** 1 Department of Genetics, Physical Anthropology and Animal Physiology, University of the Basque Country (UPV/EHU), Leioa, Basque Country Spain; 2 Biobizkaia Health Research Institute, Barakaldo, Basque Country Spain; 3 Pediatric Gastroenterology Unit, Cruces University Hospital, Barakaldo, Basque Country Spain; 4 Spanish Biomedical Research Center in Diabetes and Associated Metabolic Disorders (CIBERDEM), Madrid, Spain

**Keywords:** Celiac disease, XWAS, Mendelian randomization, *TMEM187*, Monocytes, eQTLs

## Abstract

**Background:**

Celiac disease (CeD) is an immune-mediated disorder that develops in genetically predisposed individuals upon gluten consumption. HLA risk alleles explain 40% of the genetic component of CeD, so there have been continuing efforts to uncover non-HLA *loci* that can explain the remaining heritability. As in most autoimmune disorders, the prevalence of CeD is significantly higher in women. Here, we investigated the possible involvement of the X chromosome on the sex bias of CeD.

**Methods:**

We performed a X chromosome-wide association study (XWAS) and a gene-based association study in women from the CeD *Immunochip* (7062 cases, 5446 controls). We also constructed a database of X chromosome cis-expression quantitative trait *loci* (eQTLs) in monocytes from unstimulated (*n* = 226) and lipopolysaccharide (LPS)-stimulated (*n* = 130) female donors and performed a Summary-data-based MR (SMR) analysis to integrate XWAS and eQTL information. We interrogated the expression of the potentially causal gene (*TMEM187*) in peripheral blood mononuclear cells (PBMCs) from celiac patients at onset, on a gluten-free diet, potential celiac patients and non-celiac controls.

**Results:**

The XWAS and gene-based analyses identified 13 SNPs and 25 genes, respectively, 22 of which had not been previously associated with CeD. The X chromosome cis-eQTL analysis found 18 genes with at least one *cis*-eQTL in naïve female monocytes and 8 genes in LPS-stimulated female monocytes, 2 of which were common to both situations and 6 were unique to LPS stimulation. SMR identified a potentially causal association of *TMEM187* expression in naïve monocytes with CeD in women, regulated by CeD-associated, eQTL-SNPs rs7350355 and rs5945386. The CeD-risk alleles were correlated with lower *TMEM187* expression. These results were replicated using eQTLs from LPS-stimulated monocytes. We observed higher levels of *TMEM187* expression in PBMCs from female CeD patients at onset compared to female non-celiac controls, but not in male CeD individuals.

**Conclusion:**

Using X chromosome genotypes and gene expression data from female monocytes, SMR has identified *TMEM187* as a potentially causal candidate in CeD. Further studies are needed to understand the implication of the X chromosome in the higher prevalence of CeD in women.

**Supplementary Information:**

The online version contains supplementary material available at 10.1186/s13293-023-00572-1.

## Plain language summary

Celiac disease (CeD) is an immune-related condition triggered by gluten consumption in genetically susceptible individuals. Women present higher prevalence of CeD than men, but the biological explanation of such difference has not been elucidated. In this study, we investigated whether specific genetic variations on the X chromosome were associated with CeD in each sex. Surprisingly, we found 13 genetic variants and 25 genes significantly linked to CeD in women, but not in men. Additionally, we identified genetic variants on the X chromosome associated with gene expression of monocytes, a type of immune cells that is activated in CeD after gluten intake. Integrating these data with our previous findings, we found that lower expression of a gene termed *TMEM187* might be associated with a potential increase in CeD risk in women. Finally, validation experiments confirmed higher *TMEM187* levels in blood cells from female CeD patients compared to non-celiac women, while no such difference was seen in males. In summary, our study suggests that the X-chromosome gene *TMEM187* may play a key role in CeD development, providing insights into the higher prevalence of CeD in females.

**Supplementary Information:**

The online version contains supplementary material available at 10.1186/s13293-023-00572-1.

## Highlights

- The XWAS and gene-based association study identified 13 genetic variants and 25 genes significantly associated with CeD in women, but not in men.
- eQTL analyses in naïve and LPS-stimulated female monocytes identified 16 unique genes with at least one *cis*-eQTL in the naïve condition, 6 in the LPS condition and 2 genes, namely *ZNF185* and *TMEM187*, common to both conditions.
- SMR identified three SNPs (rs7350355, rs5945386, rs80208125) associated with gene *TMEM187*. The CeD risk alleles were negatively correlated with *TMEM187* expression.
- PBMCs from active female CeD patients showed significantly higher *TMEM187* expression than controls, but no differences were observed in men.

**Supplementary Information:**

The online version contains supplementary material available at 10.1186/s13293-023-00572-1.

## Background

Celiac disease (CeD) is an immune-mediated enteropathy that develops in genetically predisposed individuals as a reaction to gluten ingestion [1]. The global prevalence of CeD is 1.4% according to serological diagnosis, and 0.7% based on biopsy confirmation [2]. As in other autoimmune diseases, the prevalence of CeD is significantly higher in women [3]. Almost all CeD patients carry the Human Leucocyte Antigen (HLA) alleles that encode the HLA-DQ2 and/or HLA-DQ8 molecules. However, HLA is necessary, but not sufficient to develop the disease, and only explains 40% of the overall genetic risk [4, 5]. GWAS and fine-mapping studies like the *Immunochip* project have identified more than 40 non-HLA *loci* associated with CeD [6–8]. Nevertheless, most of the SNPs located in these *loci* either map to non-coding regions of the genome, far away from genes, or are in strong linkage disequilibrium (LD) with other associated variants, making it difficult to identify the genes that are functionally involved in the disease [9]. Altogether, HLA and non-HLA variants identified so far explain around 50% of CeD heritability [10]. The missing genetic heritability hypothesis suggests that additional, unidentified genetic and environmental factors are involved in the development of CeD. In this sense, the X chromosome has been historically ignored in most GWAS, or has been analyzed as if it were another autosome, without accounting for male hemizygosity and female X chromosome inactivation (XCI), with only very few studies that take these considerations into account [11–13].

Several studies have shown a relationship between the risk of different autoimmune diseases, including systemic lupus erythematosus (SLE)*,* Sjögren syndrome, type 1 diabetes mellitus and CeD, and X chromosome aneuploidies like Klinefelter, Triple X or Turner syndromes [14–17]*.* Additionally, many of the approximately 1100 genes on the X chromosome are thought to be related to the immune function [18, 19]. These findings suggest a role for the X chromosome in the biased sex-prevalence of these conditions. In the case of CeD, most risk *loci* are located on the autosomes but X chromosome genes have also been identified, including *TLR7* and *TLR8, HCFC1, TMEM187* and *IRAK1* [6, 7].

Monocytes are a fundamental part of the innate immune defense against microorganisms [20]. Different studies have also related this type of immune cells to CeD, and gliadin peptides stimulate their production of IL-8 and *TNF-*α, especially in celiac patients [21, 22]. It has been suggested that the response triggered by gliadin in monocytes is similar to that induced by lipopolysaccharide (LPS) through receptors such as TLR4 [23]. Fairfax et al*.* analyzed how genetic variants shape gene expression in monocytes, under different in vitro stimuli, including LPS, and showed that more than half of the monocyte eQTLs are specific of the environmental stimulus, but the X chromosome was not included in the analysis [24].

Mendelian randomization (MR), and more specifically the Summary-data-based MR (SMR), integrates GWAS and eQTL summary statistics in order to detect the functional involvement of genes under the GWAS peaks [25]. Particularly, association hits are translated into potentially causal relationships between expression levels of candidate genes and complex traits, in relevant tissues, cell types and context. Again, previous analyses in CeD that have made use of this method to combine GWAS and eQTL summary statistics have omitted the X chromosome [26, 27].

In this study, we hypothesized that the X chromosome could harbor additional susceptibility *loci* that could help explain both the missing heritability and the higher prevalence in women. Therefore, we aimed to identify genes on the X chromosome that might participate in the pathogenesis and also contribute to the sex bias in CeD, through their specific transcriptional profile in monocytes. For that purpose, we performed an X-chromosome association study (XWAS) in women from the *Immunochip* project, and constructed a database of X chromosome *cis*-eQTLs in female monocytes. Finally, we combined the two datasets using SMR in order to find monocyte-specific functional candidates on the X chromosome involved in CeD*.*

## Materials and methods

### *Immunochip* data and X chromosome association analyses

The CeD *Immunochip* dataset was filtered to include only X chromosome variants with genotyping rate > 95%, minor allele frequency (MAF) > 1% and *P*-value from Hardy Weinberg equilibrium (*P*_HWE_) test > 1 × 10^–6^ using PLINK1.9 [28]. We removed individuals with call rate < 97% and heterozygosity deviating more than 4 standard deviations from the mean (> 4SD). Genotypes were imputed at the Michigan Imputation Server [29] using HRC r1.1 2016 (GRCh37/hg19) as a reference panel, using only the European population, Minimac4 as the imputation software and phasing with Eagle v2.4. Imputed SNPs with an *R*^2^ imputation accuracy above 0.8, MAF > 1% and *P*_HWE_ > 1 × 10^–6^ were kept. After applying these filters, 12,508 female samples (7062 cases and 5446 controls) and 9474 males (3712 cases and 5762 controls) and 1611 SNPs were retained. We calculated the first ten principal components of the genotypes using PLINK1.9 to control for potential population stratification in downstream analyses. The top associated SNP was replicated in the Dubois et al*.* study GWAS dataset [6].

The CeD XWAS was performed separately for men and women using the newml method implemented in SNPTEST (version 2.5.6), assuming a complete inactivation of one chromosome in females and equal effect size in both sexes [30]. Specifically, this method uses a logistic regression model encoding genotypes in males as 0/1 and in females as 0 / ½ / 1. The analysis was performed assuming an additive genetic model and the first ten principal components of the genotype data were included as covariates. The *P*-value threshold for statistical significance was set at *P* < 8.68 × 10^–5^ after Bonferroni correction according to the number of independent tests, as determined with the SimpleM method [31]. Results were plotted on a Manhattan plot generated with the qqman R package [32].

A gene-based association analysis was carried out in women using the FastBAT method available in the Genome-wide Complex Trait Analysis (GCTA) software package [33, 34]. This method integrates GWAS summary statistics and LD information to calculate the *P*-value of a set of variants within a preset distance from a gene. The analysis was performed using the default settings suggested by GCTA-FastBAT: gene regions extended 50 kb away from both the 3′ and 5′ UTRs of the genes, and SNPs in strong LD (*r*^2^ ≥ 0.9) were pruned. We conducted the analysis for 2393 genes and 1611 SNPs and the *P*-value significance threshold was set at *P* < 0.05 after False Discovery Rate (FDR) correction. A regional association plot combining XWAS and gene-based association results was generated with an open-source R script (https://github.com/Geeketics/LocusZooms).

### Monocyte *cis*-eQTLs

A catalog of naïve and LPS-stimulated monocyte *cis*-eQTLs of the X chromosome was constructed in women using SNP genotype and expression data from a general population study by Fairfax et al*.* [24]. X chromosome variants and individuals were filtered and imputed as described above. Additionally, only those SNPs that were homozygous for the minor allele in at least 5 samples were retained. After the QC, 233 female samples and 165,648 variants remained for subsequent analyses.

Monocyte expression data that had already undergone data normalization, transformation with Variance Stabilizing Transformation (VST), batch effect correction and removal of outliers, were subjected to additional QC steps using the IluminaHumanv4.db [35] to remove those probes that matched more than one *locus*, those on the autosomes, Y chromosome or without chromosome reported, those containing SNPs with MAF > 0.1, and those described as *Bad* or *No match*. This resulted in 1347 probes mapping to 1250 stable gene IDs in the X chromosome.

We combined genotype (165,648 SNPs) and gene expression (1250 genes) information of 226 samples from the naïve monocyte female dataset using the QTLtools software [36]. The associations between SNP genotypes and gene expression levels were tested with simple linear regressions assuming normal distribution of the data, and the first 10 principal components of the genotype were included as covariates. Only SNPs within a 1-Mb window from the transcription start site (TSS) of a gene were analyzed. P_eQTL_ values were calculated with the nominal pass option and a *P*_eQTL_ < 5 × 10^–8^ threshold was set to identify significant results. The same analysis was performed with expression data from LPS-stimulated monocytes in women (*n* = 130).

### SMR analyses

SMR was performed combining the summary statistics from the XWAS and the eQTLs of naïve and LPS-stimulated monocytes using the SMR software [25]. Briefly, SMR uses cis-eQTLs as instrumental variables, gene expression as the exposure, and CeD as the outcome, to infer genes pleiotropically or causally associated with CeD. SMR results were subjected to the heterogeneity in dependent instruments (HEIDI) test to detect the presence of LD. In this test, a significant *P*-value suggests that the association detected could be the result of two genetic variants in strong LD, whereas a non-significant *P*-value indicates that a single variant is associated with both gene expression and the disease. We used the following default parameters suggested by SMR: the *cis*-window was set at 2 Mb, the threshold *P*_eQTL_ for the SMR analysis was set at 5 × 10^–8^, the threshold *P*_eQTL_ for the HEIDI test was set at 1.57 × 10^–3^ and SNPs with a LD *r*^2^ > 0.9 or *r*^2^ < 0.05 with the top associated eQTL were pruned. SMR results with an FDR *q*-value < 0.05 and *P*_HEIDI_ > 0.05, were considered as pleiotropic or causal associations. We plotted the *TMEM187* region (2000 kb) on the X chromosome using the *SMRLocusPlot* script available in the SMR website.

### Expression analysis in pediatric CeD patients

*TMEM187* expression was quantified in peripheral blood mononuclear cells (PBMCs) from CeD children at onset (20 females, 8 males), patients on gluten-free diet (GFD) (6 females, 9 males), potential CeD patients (5 females, 6 males) and non-celiac controls (17 females, 10 males). CeD was diagnosed at the Pediatric Gastroenterology Unit of Cruces University Hospital. The study was approved by the Clinical Research Board of Cruces University Hospital. Samples (2.5 ml in EDTA-containing tubes) were collected after informed consent had been obtained from their parents or guardians and transferred to the Basque Biobank for Research. PBMCs were isolated using the MACSprep™ PBMC Isolation kit (Miltenyi Biotec SL, Madrid, Spain; cat. no. 130–115-169), RNA was purified using the NucleoSpin® RNA mini kit (Macherey–Nagel, Düren, Germany; cat. no. 740955.250 4,392,653) and stored at – 80 °C until use.

The expression of *TMEM187* was quantified by RT-qPCR using the TaqMan RNA-to-Ct 1-Step Kit (Thermo Fisher Scientific Inc., Waltham, MA, USA; cat. no. 4392653) and a commercially available TaqMan Gene Expression assay (Thermo Fisher Scientific Inc., cat. no. Hs01920894_s1) on a Bio-Rad CFX Real Time PCR system (Bio Rad Scientific, Hercules, CA, USA). The housekeeping gene *RPLPO* was simultaneously measured and used as an endogenous control. Relative expression in each sample was calculated using the 2^−ΔΔCt^ method and therefore, the expression of each sample was normalized to both the expression of *RPLPO* and the average of the controls. Differences in gene expression levels were analyzed with Mann–Whitney U-test using GraphPad Prism v.8.0.1. Finally, the expression of *TMEM187* in different immune cell types (B, NK, T cells, and monocytes) was analyzed and sex-stratified expression plots were constructed using the default settings of the Database of Immune Cell Expression eQTL Epigenomics (DICE, https://dice-database.org/) online browser [37].

## Results

### XWAS and gene-level association analyses in CeD

To determine whether the sex bias in CeD prevalence is related to the X chromosome, we performed the XWAS of CeD in women and men separately (Additional file 1). We identified a single association peak on Xq28 including 13 significant SNPs (*P*_XWAS_ < 8.68 × 10^–5^), with rs78237385 (*P*_XWAS_ = 2.30 × 10^–5^, OR = 1.20 ± 0.10) as the top SNP (Additional file 2). The male XWAS did not detect any significant association (Additional file 3). The top SNP was also suggestively associated with CeD in females from the Dubois et al. study (*P*-value = 1.86 × 10^–4^) but was not significant (*P*-value = 0.19) in men from the same study (Additional file 4).

**Fig. 1:**
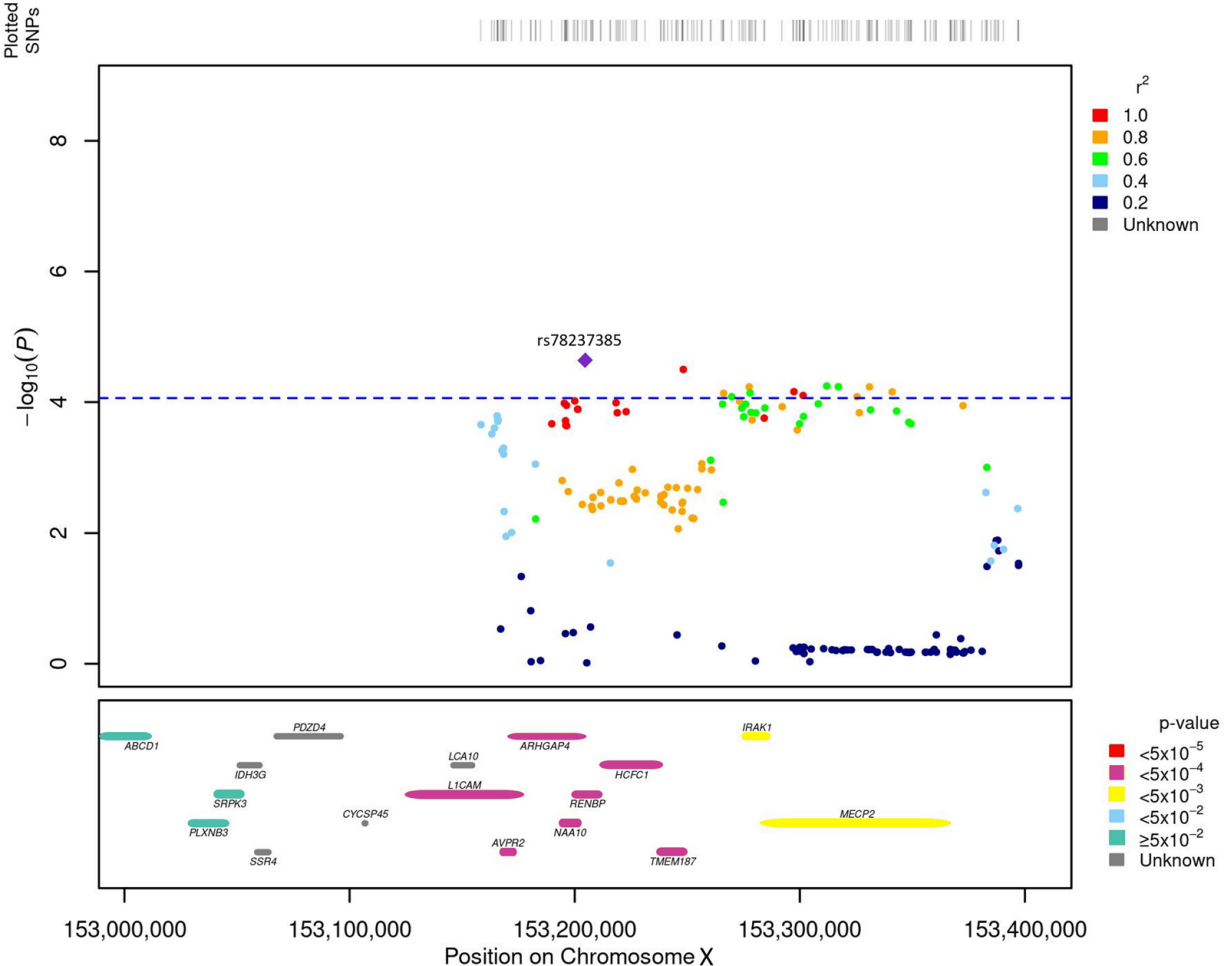
Locus zoom plot 200 kb upstream and downstream of the XWAS top SNP. The XWAS top SNP, rs78237385 is represented with a purple diamond. In the top panel, the color schema represents the LD between the top SNP and the SNPs included in the region. In the bottom panel, the color schema represents the P-value from the gene-based association analysis

We also performed a gene-based association analysis in women, that takes into account the aggregated effect of sets of SNPs. We introduced 2392 genes and 1611 SNPs in the analysis and defined each gene region as ± 50 kb from both 3′ and 5′ UTRs. As a result, 276 association tests were performed and 25 candidate genes in the X chromosome were identified to be associated with CeD in women (FDR *q*-value < 0.05) (Additional file 5). Out of the 25 genes, 22 are novel genes associated with CeD, although *ARHGAP4*, *RENBP*, *NAA10*, *AVPR2* or *MECP2* had been previously identified in other autoimmune diseases [38–40]. XWAS and gene-level analysis results are summarized in Fig. 1.

### Cis-eQTL analysis of the X chromosome in monocytes of women

In order to obtain an additional layer of information to interpret the XWAS signal, we calculated the X chromosome eQTLs in monocytes from women. We analyzed 226 samples with informative genotype and gene expression data from naïve monocytes, and performed multiple linear regressions between 165,648 SNPs and 1250 probes corresponding to 819 genes. Applying a threshold *P*_eQTL_ value of 5 × 10^–8^, we identified 1097 *cis*-eQTLs involving 1054 SNPs, 19 probes and 18 independent genes (Additional file 6). The analysis in 130 LPS-stimulated female monocyte samples revealed 150 cis-eQTLs, corresponding to 94 SNPs, 9 probes and 8 genes (Additional file 7). Two genes (*ZNF185* and *TMEM187*) were common to both situations, 16 were unique to naïve female monocytes, and 6 to female monocytes after LPS stimulation.

### XWAS and cis-eQTL SMR and gene expression analyses

**Fig. 2:**
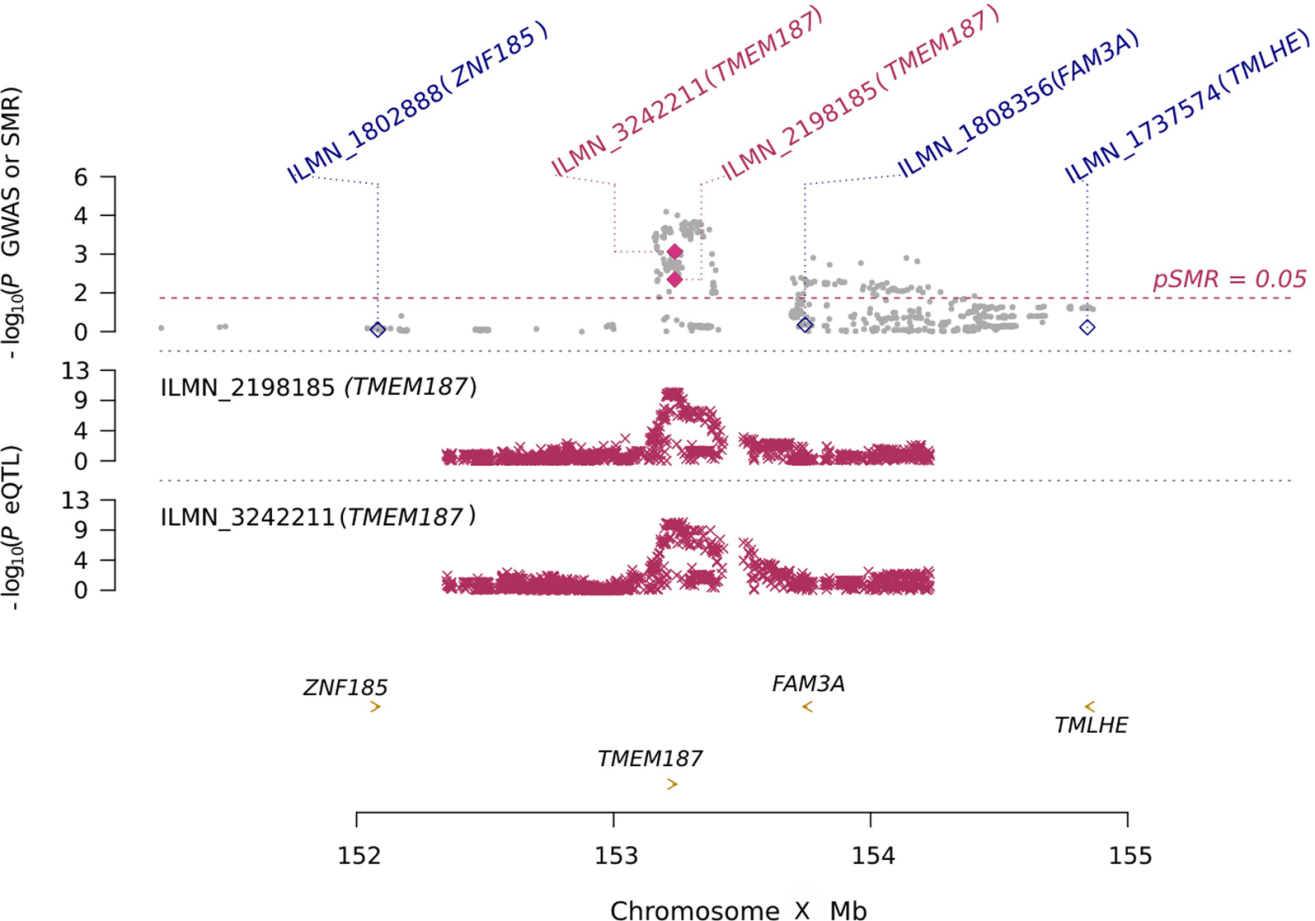
SMR *locus* plot of the results of the SMR analysis between the CeD XWAS and the naïve female monocyte eQTLs. In the top panel, grey dots represent − log_10_(*P*-values) for the female XWAS SNPs. Diamonds represent − log_10_(*P*-values) for probes from the SMR analysis and filled diamonds show those that pass the HEIDI test. In the middle panel, the red crosses represent − log_10_(*P*-values) for gene probes in the eQTL analysis. In the bottom panel, the location of the probes on the X chromosome is shown

The SMR and HEIDI analyses of the summary statistics of the female CeD XWAS and the naïve monocyte *cis*-eQTL dataset identified two SNPs (rs7350355 and rs5945386) that were associated with two expression probes (ILMN_2198185 and ILMN_3242211, respectively) corresponding to the same gene, *TMEM187*, with a *q*-value < 0.05 and *P*_HEIDI_ > 0.05. The minor alleles rs7350355*G and rs5945386*G are both the CeD risk alleles and were negatively correlated with *TMEM187* expression (Fig. 2; Additional file 8). The SMR analysis was replicated with *cis*-eQTLs from LPS-stimulated female monocytes and revealed a single SNP (rs80208125) that was associated with the same two *TMEM187* probes (Additional file 9; Additional file 10).

**Fig. 3:**
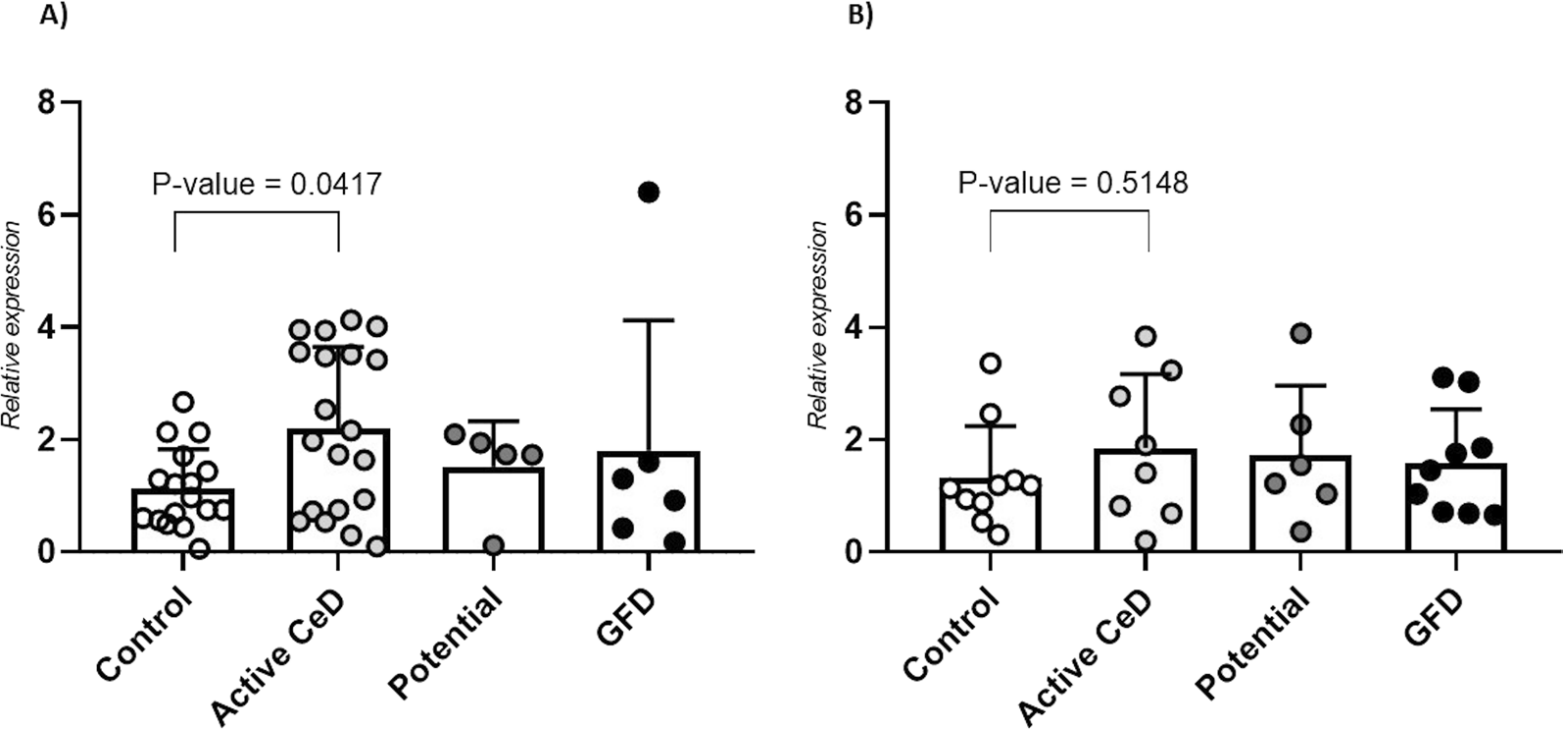
Results of the expression analysis. **A** Expression of *TMEM187* in PBMCs of female samples. **B** Expression of *TMEM187* in PBMCs of male samples. Both female and male samples were classified into four groups: non-celiac controls, celiac patients at onset, potential celiac patients and celiac patients on GFD represented by white, light grey, dark grey and black circles, respectively

We investigated the expression of *TMEM187* in PBMCs from female CeD patients at diagnosis, on GFD, potential CeD patients and non-celiac female controls. *TMEM187* showed a significantly higher expression in active CeD patients compared to controls (*P*-value = 0.0417) and no differences were observed in potential CeD and GFD-treated individuals (Fig. 3A). We also studied *TMEM187* expression in men but no significant differences were found (Fig. 3B). On the other hand, the expression of *TMEM187* varied among different immune cell types (Additional file 11).

## Discussion

The prevalence of CeD is significantly higher in women, as in the case of other autoimmune diseases [3]. A higher prevalence of immune-mediated disorders has also been observed in individuals with syndromes related to X chromosome aneuploidies [14, 17], suggesting an implication of the X chromosome in the risk of autoimmune diseases, probably also in the risk of CeD. In the current study, we have focused on gene expression in female monocytes in order to identify genes on the X chromosome that are involved in CeD and could explain the sex bias in the disease prevalence. For that purpose, we conducted a sex-specific XWAS in women from the *Immunochip* dataset and identified 25 genes associated with CeD in women, of which 22 have not been previously reported. Remarkably, 24 out of the 25 genes that were significant in the analysis are located on chromosome region Xq28, which has been previously associated with different autoimmune conditions such as SLE [39], rheumatoid arthritis [40], systemic sclerosis [38] or CeD [7].

On the other hand, it has been suggested that gliadin triggers an innate response in monocytes similar to that produced by LPS [23]. We performed an X chromosome *cis*-eQTL analysis in female naïve and LPS-stimulated monocytes and identified 6 eQTL-genes unique to LPS-stimulated female monocytes, some of which have been previously associated with autoimmune disorders, including *PLXNA3* [41].

We then integrated the XWAS with cis-eQTL data from female naïve and LPS-stimulated monocytes using an MR approach. To our knowledge, this is the first SMR analysis of the X chromosome in CeD. We identified two SNPs (rs7350355 and rs5945386) that regulate the expression of *TMEM187* in naïve monocytes and a different SNP (rs80208125) associated with *TMEM187* in LPS-stimulated monocytes, suggesting that the genetic background may be more important than LPS stimulation in the regulation of *TMEM187* expression. rs7350355 is a missense variant located within exon 2 of *TMEM187*, rs5945386 is an intergenic variant located 21 kb downstream of *TMEM187,* and rs80208125 is located on the 5’ UTR of *TMEM187*. All these variants exhibit strong LD (> 0.87) in the British population in England and Scotland, from the 1000 Genomes Project [42]. In all three cases, the CeD risk alleles (rs7350355*G, rs5945386*G and rs80208125*G) were the minor alleles (MAF around 0.2) and were negatively correlated with *TMEM187* expression. *TMEM187* encodes a multipass transmembrane protein of unknown function [43], and it has been proposed as a putative candidate gene in CeD together with *IRAK1* and *HCFC1,* located in the same *locus* [7]. As far as we know, the only study that observed *TMEM187* dysregulation in CeD is the one carried out by Pascual et al. in duodenal biopsies [44]. The *TMEM187 locus* has also been associated with other autoimmune disorders such as SLE or rheumatoid arthritis [39, 45], supporting the hypothesis of a shared genetic background in autoimmune disorders.

In our study, we interrogated the expression of *TMEM187* in PBMCs from celiac patients at onset, celiac patients on GFD, potential celiac patients and non-celiac controls. PBMCs are a mixture of immune cells that contain monocytes (10–20%), together with lymphocytes (70–90%) and dendritic cells (1–2%), among others [46]. We observed a higher expression of *TMEM187* in PBMCs from female pediatric patients at disease onset compared to non-celiac children. These results are consistent with a study published in 2016 by Pascual et al. that showed an upregulation of *TMEM187* expression in biopsies of celiac adults [44]. The upregulation of *TMEM187* in female CeD patients was not replicated in male PBMCs, suggesting possible role of *TMEM187* in the sex bias of CeD that nevertheless needs to be confirmed with additional investigations.

The overexpression reported for *TMEM187* in female CeD patients at onset is apparently contradictory to the fact that CeD risk alleles correlate with lower expression. This observed divergence could be due to different reasons: first, the CeD risk eQTLs could have an effect at the protein level, taking into account that rs7350355 is a missense variant that could alter the function of TMEM187, regardless of mRNA quantity. On the other hand, we are unable to definitively ascertain which of the eQTLs is the causal SNP, given the strong LD. Another reason could be that PBMCs contain a relatively modest proportion of monocytes, and the SNP could have different effects on the gene expression in other cell types, therefore explaining the apparent contradiction. The highly variable expression of *TMEM187* in different immune cell types warrants further research on its role in the immune system. In addition, we have to bear in mind that the present expression analyses have been carried out in a pediatric cohort of diagnosed celiac children, while female donors in the monocyte expression study were non-celiac adults. It has been well reported that disease and immunogenic insult can sometimes surpass the genotypic effect, and lead to this kind of apparently contradictory situations [47]. Finally, Pascual et al*.* observed differences in the gene expression profile of susceptibility genes in CeD between children and adults, including *TMEM187* [44].

Last but not least, it is worth mentioning that the main objective in the present work was not to identify SNPs with a functional involvement in CeD, nor to ascertain the mechanism by which they exert their function, but to highlight potentially causal genes that participate in the pathogenesis of the disease through their expression in monocytes. We carried out both the XWAS and the eQTL calculations with the aim of obtaining instruments to perform downstream analyses such as SMR, being aware that in our results SNPs will lose their relevance and will be replaced by functional candidate genes.

One limitation of this study is that both the lack of association as well as the absence of significant differential expression between CeD cases and controls observed in men could be due to the smaller sample size of the male cohort. This is partly a consequence of the higher incidence of CeD in women and an important factor to consider. However, we have studied the association of the top SNP of our female XWAS (rs78237385) in an independent dataset [6] and the *P*-values in men and women are 0.19 and 1.86 × 10^–4^, respectively. Additionally, the SNPs with significant results in the SMR analysis (rs5945386, rs7350355 and rs80208125) have *P*-values in women of 8.29 × 10^–5^, 4.66 × 10^–3^ and 3.34 × 10^–3^, respectively, while in men, they show *P*-values of 0.50, 0.49 and 0.57, respectively. We consider these *P*-values unlikely to become significant even with a higher number of male samples, and this lack of a significant association in men could imply a possible divergent mechanism of pathogenesis between sexes, that could explain the increased prevalence of CeD, and other autoimmune diseases observed in women [48].

## Perspectives and significance

This is the first SMR approach in the X chromosome in CeD. We have identified *TMEM187* as a candidate gene in CeD in monocytes and validated its differential expression in PBMCs from female CeD patients at onset. The fact that both the genetic association and the differential gene expression are not found in male patients suggests a role for *TMEM187* in the sex bias observed in CeD. SMR appears as a useful approach to identify potentially causal genes under association peaks, including the X chromosome. Further studies are needed to identify the function of *TMEM187* and understand its behavior in different cell types and disease status, and to clarify its role in CeD pathogenesis and the sex bias.

### Supplementary Information

**Additional file 1.** Sex-specific summary statistics from *Immunochip* XWAS in women and men. BP: position of the variant; OA: other allele; EA: effect allele; F: frequency of the effect allele; B: beta value, SE: standard error of the beta, P: P-value.

**Additional file 2.** Manhattan plot of the CeD XWAS in women. The top SNP rs78237385 (P-value = 2.30 × 10^−5^) is shown as a red circle. The blue line represents the significant threshold according to the Bonferroni correction for the number of independent tests (P-value = 8.68 × 10^−5^).

**Additional file 3.** Manhattan plot of the CeD XWAS for CeD in men. The blue line represents the significant threshold according to the Bonferroni correction for the number of independent tests (P-value = 8.68 × 10^−5^).

**Additional file 4.** Nominal P-values of the top *Immunochip* female XWAS SNP (rs78237385) in the different datasets stratified by sex. EAF: frequency of the effect allele in cases; EAF: frequency of the effect allele in controls; OR: odd ratio.

**Additional file 5.** CeD candidate genes on the X chromosome identified by gene-based association analysis at q-value < 0.05. Top associated SNP: the top associated XWAS SNP in the region; Top PXWAS: P-value of the top associated XWAS SNP in the region; PfastBAT: gene-based test P-value; q-value: FDR adjusted gene-based test P-value.

**Additional file 6.** cis-eQTLs identified on the X chromosome by cis-eQTL analysis of naïve female monocytes at nominal P-value < 5 × 10^–8^. A total of 1,097 cis-eQTLs, implicating 1,054 SNPs, 19 probes and 18 genes were identified on the X chromosome of naïve female monocytes. n_var_in_cis: the total number of variants tested in cis; dist_gene_var: the distance between the gene and the tested variant; var_position: the position of the variant; nom_pval: the nominal P-value of the association between the variant and the gene; r_squared: the correlation coefficient of the linear regression; slope: the slope (beta) of the linear regression; slope_se: the standard error of the beta.

**Additional file 7.** cis-eQTLs identified in the X chromosome by cis-eQTL analysis of LPS-stimulated female monocytes at nominal P-value < 5 × 10^–8^. A total of 150 cis-eQTLs, implicating 94 SNPs, 9 probes and 8 genes were identified on the X chromosome of LPS-stimulated female monocytes. n_var_in_cis: the total number of variants tested in cis; dist_gene_var: the distance between the gene and the tested variant; var_position: the position of the variant; nom_pval: the nominal P-value of the association between the variant and the gene; r_squared: the correlation coefficient of the linear regression; slope: the slope (beta) of the linear regression; slope_se: the standard error of the beta.

**Additional file 8.** Summary of the SMR analysis between the CeD XWAS and the naïve female monocyte eQTLs. A1: the effect allele; A2: the other allele; b_XWAS: the effect size from XWAS; p_XWAS: P-value from the XWAS; b_eQTL: the effect size from eQTL analysis; p_eQTL: P-value from the eQTL analysis; b_SMR: the effect size from the SMR analysis; p_SMR: nominal P-value from the SMR analysis; q-value: FDR adjusted P-value. Results in bold indicate statistical significance after multiple testing correction.

**Additional file 9.** SMR *locus* plot of the results of the SMR analysis between the CeD XWAS and the LPS-stimulated female monocyte eQTLs. In the top panel, grey dots represent -log_10_(P-values) for the female XWAS SNPs. Diamonds represent -log_10_(P-values) for probes from the SMR analysis and filled diamonds show those that pass the HEIDI test. In the middle panel, the red crosses represent -log_10_(P-values) for gene probes in the eQTL analysis. In the bottom panel, the location of the probes on the X chromosome is shown.

**Additional file 10.** Summary of the SMR analysis between the CeD XWAS and the LPS-stimulated female monocyte eQTLs. A1: the effect allele; A2: the other allele; b_XWAS: the effect size from XWAS; p_XWAS: P-value from the XWAS; b_eQTL: the effect size from eQTL analysis; p_eQTL: P-value from the eQTL analysis; b_SMR: the effect size from the SMR analysis; p_SMR: nominal P-value from the SMR analysis; q-value: FDR adjusted P-value. Results in bold indicate statistical significance after multiple testing correction.

**Additional file 11.** *TMEM187* expression in different immune cells. Red and blue boxes represent *TMEM187* expression in the different immune cells from females and males, respectively.

## Data Availability

The significant results of XWAS, gene-based association analysis, eQTL analysis, and SMR analysis are included article and its additional files. The complete summary statistics generated during the current study are available from the corresponding author on reasonable request. Monocyte data were obtained from public repositories, according to a Data Transfer Agreement between the University of the Basque Country (UPV/EHU) and the University of Oxford. The genotyping data were downloaded from the European Genome-phenome Archive (https://www.ebi.ac.uk/ega/datasets/; experiment EGAS00000000109) and the gene expression microarray data from the same individuals were downloaded from the EBI ArrayExpress database (https://www.ebi.ac.uk/arrayexpress/experiments/; experimentnumber E-MTAB-2232). This study makes use of the *Immnochip* data generated by the Wellcome Trust Case–Control Consortium (WTCC data sets EGAD00010000246, EGAD00010000248 and EGAD00010000250). A full list of the investigators who contributed to the generation of the data is available from http://www.wtccc.org.uk. Funding for the project was provided by the Wellcome Trust under awards 076113, 085475 and 090355.
