# Supplementary figures and images for: Sex bias in celiac disease: XWAS and monocyte eQTLs in women identify TMEM187 as a functional candidate gene
Source: Biol Sex Differ. 2023 Dec 11;14:86. doi: 10.1186/s13293-023-00572-1 (PMC10712119; doi:10.1186/s13293-023-00572-1)

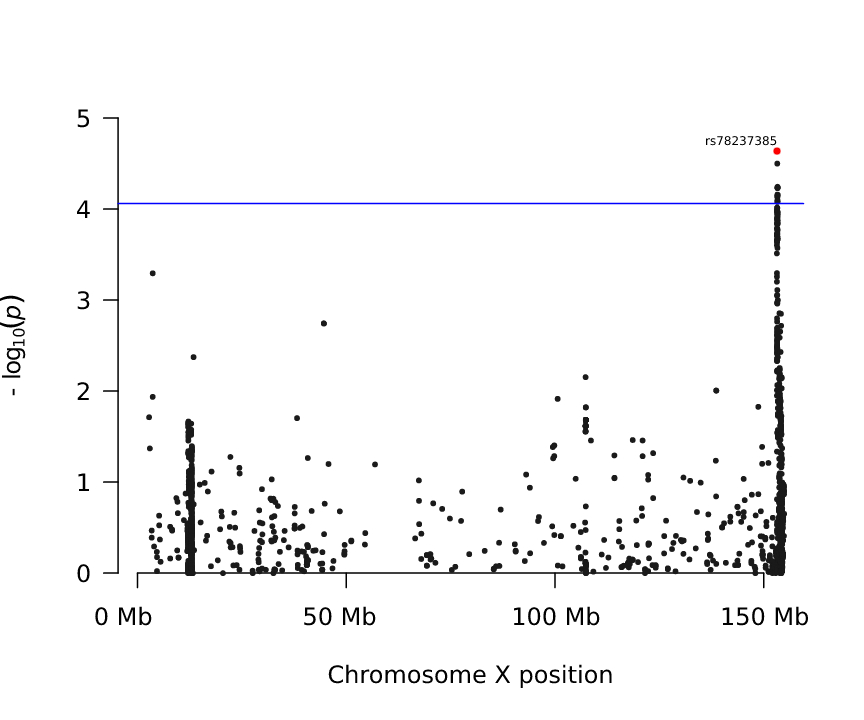

Supplement: Supplementary file 2 — Additional file 2. Manhattan plot of the CeD XWAS in women. The top SNP rs78237385 (P-value = 2.30 × 10^−5^) is shown as a red circle. The blue line represents the significant threshold according to the Bonferroni correction for the number of independent tests (P-value = 8.68 × 10^−5^). [file 13293_2023_572_MOESM2_ESM.jpg]

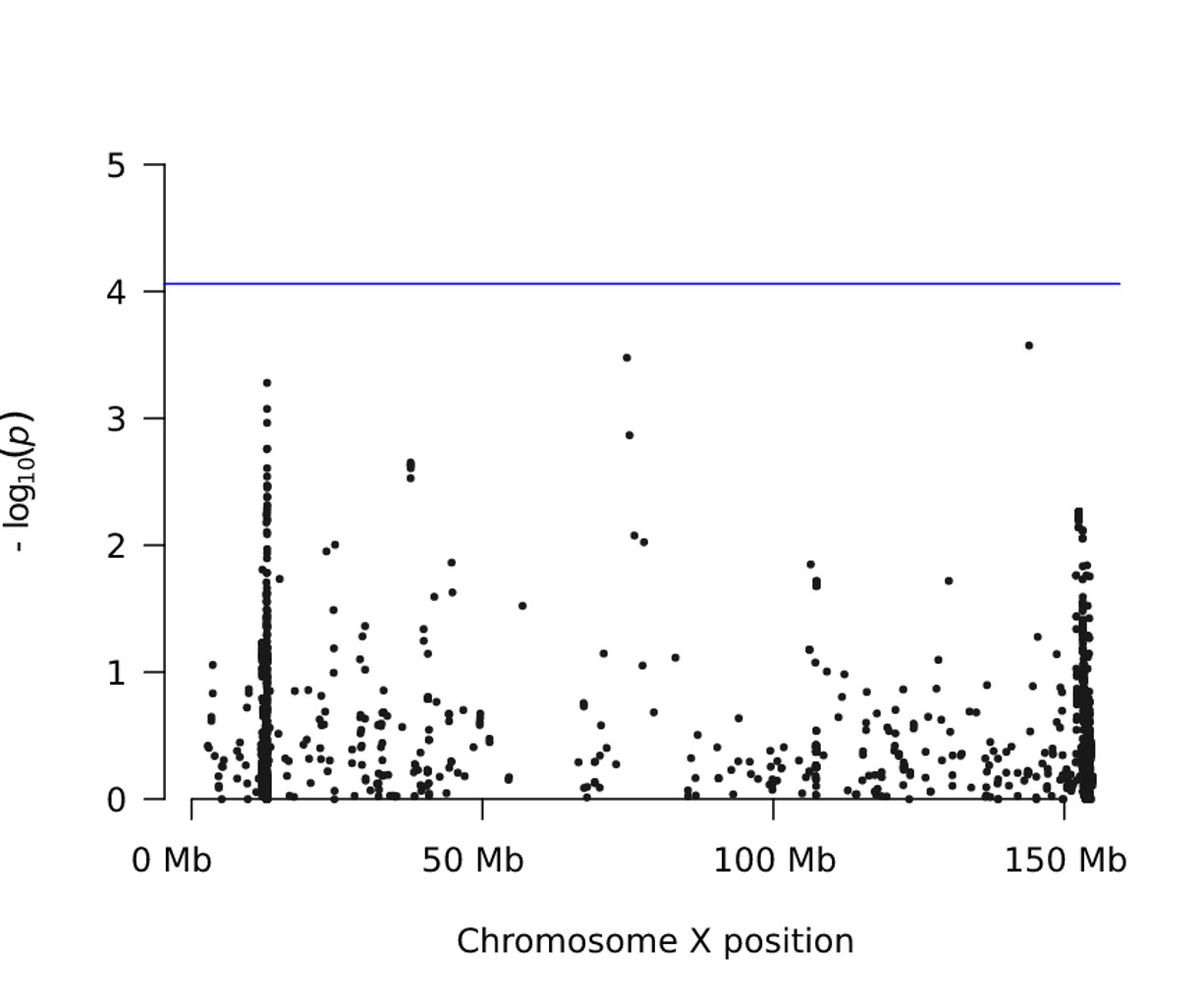

Supplement: Supplementary file 3 — Additional file 3. Manhattan plot of the CeD XWAS for CeD in men. The blue line represents the significant threshold according to the Bonferroni correction for the number of independent tests (P-value = 8.68 × 10^−5^). [file 13293_2023_572_MOESM3_ESM.jpg]

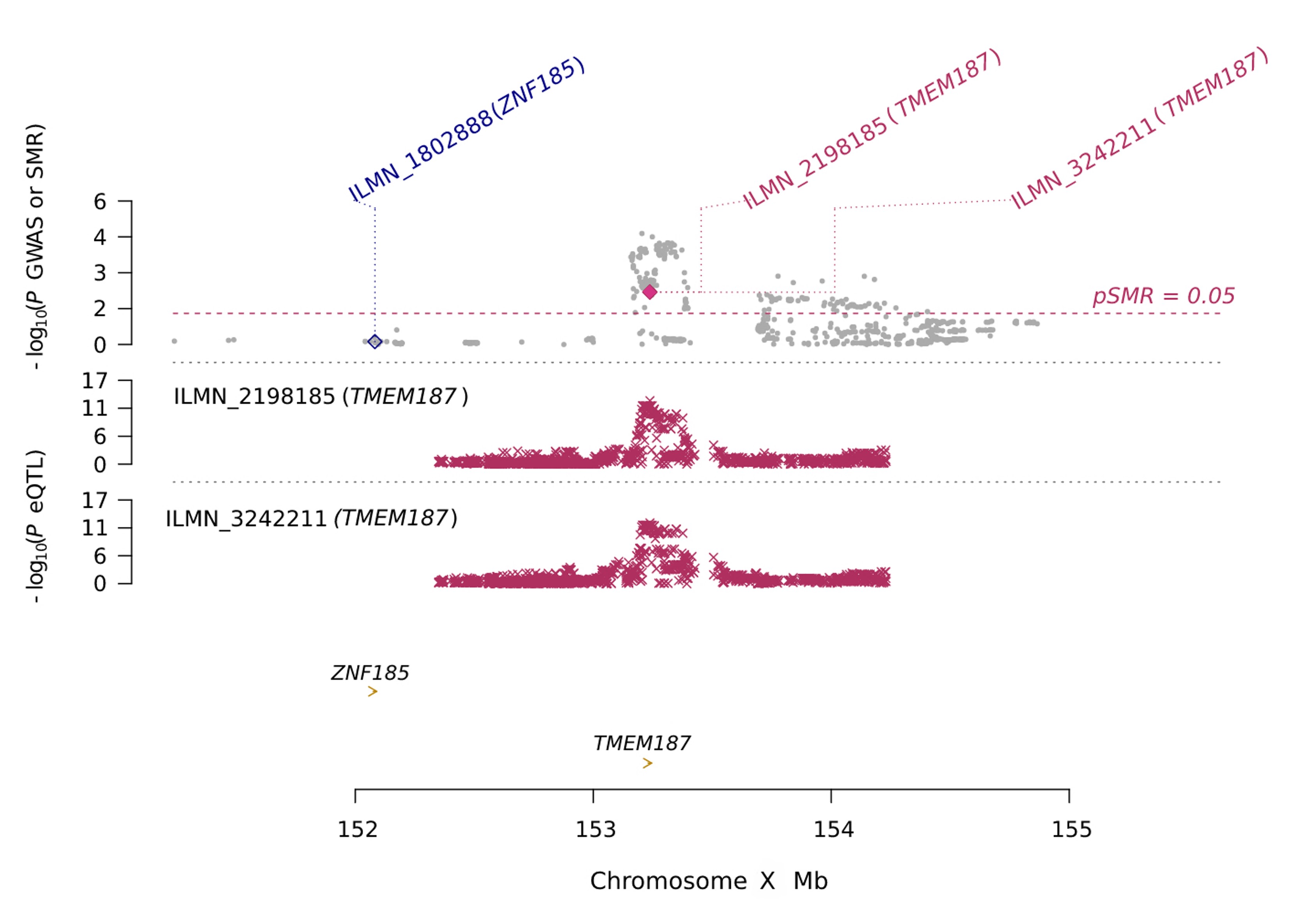

Supplement: Supplementary file 9 — Additional file 9. SMR locus plot of the results of the SMR analysis between the CeD XWAS and the LPS-stimulated female monocyte eQTLs. In the top panel, grey dots represent -log10(P-values) for the female XWAS SNPs. Diamonds represent -log10(P-values) for probes from the SMR analysis and filled diamonds show those that pass the HEIDI test. In the middle panel, the red crosses represent -log10(P-values) for gene probes in the eQTL analysis. In the bottom panel, the location of the probes on the X chromosome is shown. [file 13293_2023_572_MOESM9_ESM.jpg]

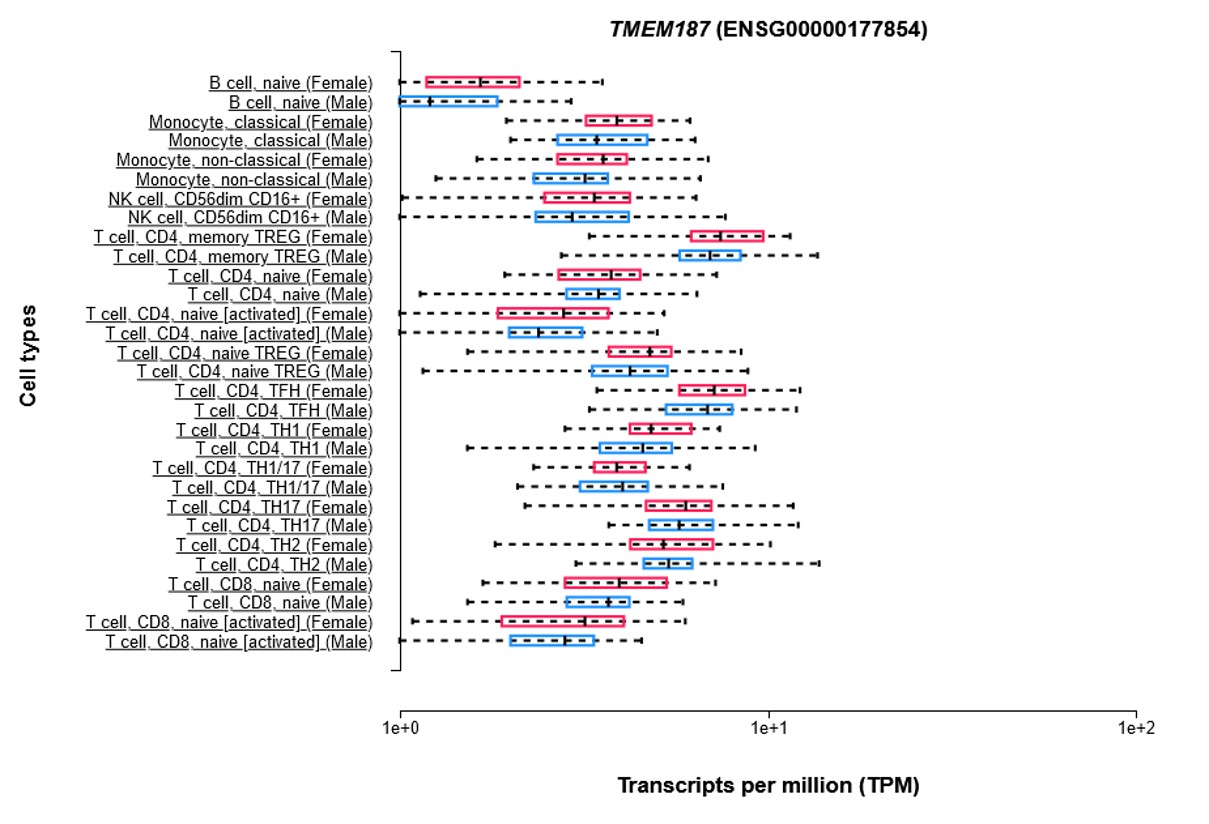

Supplement: Supplementary file 11 — Additional file 11. TMEM187 expression in different immune cells. Red and blue boxes represent TMEM187 expression in the different immune cells from females and males, respectively. [file 13293_2023_572_MOESM11_ESM.jpg]
